# Expression of OPN3 in acral lentiginous melanoma and its associated with clinicohistopathologic features and prognosis

**DOI:** 10.1002/iid3.438

**Published:** 2021-05-06

**Authors:** Wen Zeng, Wei Zhang, Jianglong Feng, Xiaoyan He, Hongguang Lu

**Affiliations:** ^1^ Department of Dermatology Affiliated Hospital of Guizhou Medical University Guiyang Guizhou China; ^2^ Department of Immunology, Basic Medical School Guizhou Medical University Guiyang Guizhou China; ^3^ Department of Pathology Affiliated Hospital of Guizhou Medical University Guiyang Guizhou China; ^4^ Department of Pathology Affiliated Cancer Hospital of Guizhou Medical University Guiyang Guizhou China

**Keywords:** acral lentiginous melanoma, foot, inguinal lymph node, junctional melanocytic nevi, melanoma, metastasis, OPN3, prognosis

## Abstract

**Background:**

OPN3 upregulation associated with metastasis was recently described in two subtypes of lung cancers. And OPN3 identified in light‐independent functions in epidermal melanocytes has already shown promise. However, in malignant melanocytic tissues, the expression and characterization of OPN3 remain uncharacterized.

**Objectives:**

We investigated the clinico‐histopathologic features in relation to OPN3 expression of acral lentiginous melanoma (ALM), which is a rare cutaneous melanoma subtype and not associated with prior sunlight exposure.

**Methods:**

In all, 84 samples of junctional melanocytic nevi (JMN, *n* = 12), primary ALMs (*n* = 39) and inguinal lymph node metastasis (ILNM, *n* = 23) from ALMs were evaluated for the immunohistochemical expression of OPN3. OPN3 messenger RNA and protein level were further determined in melanocytic tumors using quantitative real‐time PCR, multiimmunofluorescence and Western blot assays. We also estimated the associations OPN3 expression between clinicopathological features and prognosis.

**Results:**

ILNMs, in contrast to JMN and ALMs, had higher OPN3 expression scores (*p* < .001) by immunohistochemistry analysis. High OPN3 score was associated with presence of ulceration, increased Breslow depth and Clark level (*p* = .025, *p* = .042, and *p* = .012, respectively). Furthermore, a remarkable difference (*p* = .037) of patient overall survival was found when comparing the OPN3 expression of immunohistochemical score between equal to or larger than 100 and below 100 groups. Also, Cox regression models showed that high OPN3 scores were associated with worse melanoma survival.

**Conclusion:**

High OPN3 expression is significantly associated with ALMs and metastatic phenotype as well as a poor prognosis.

## Graphical abstract

Acral lentiginous melanoma has a poor prognosis due to delayed diagnosis aggressive biological behavior. However, its molecule features associated with prognosis remains largely unknown. We have detected the expression characteristic of OPN3 in ALMs, and demonstrated that high OPN3 is a negative prognostic indicator.

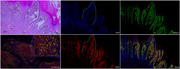

## What's already known about this topic?

Acral lentiginous melanoma has a poor prognosis due to delayed diagnosis and aggressive biological behavior in early stage. However, its molecule features associated with prognosis remains largely unknown.

## What does this study add?

We have detected the expression characteristic of OPN3 in acral lentiginous melanomas, and demonstrated that high OPN3 is a negative prognostic indicator.

## 1 INTRODUCTION

Acral lentiginous melanoma (ALM) is a rare cancer that derives from the melanocytes present within acral areas, including the palms, soles, and subungual region.^1^, ^2^ The most common site of ALM is the plantar area as a distinct subtype of cutaneous melanoma.^3^ Compared to other cutaneous melanoma subtypes, ALM has distinct epidemiological and histological characteristics, which is less associated with sun exposure, pre‐existing nevi, and family history of melanoma.^1^, ^2^, ^3^, ^4^ It mainly occurs in darker‐skinned populations such as Asia, Latin America, Africa, and Hispanic.^4^, ^5^ Histologically, large atypical melanocytes of ALM proliferate along the dermoepidermal junction in broad lentiginous growth pattern.^3^ Also, the molecular features are notably different from lentigo maligna melanoma and superficial spreading melanoma. For instance, the frequency of *BRAF* and *KIT* mutations in ALM was only 15%–25.5% and 10%–20%, respectively.^3^, ^6^ Moreover, recent reports have shown that ALM was less susceptible to anti‐PD‐1 (programmed cell death‐1) therapy, in part because of lacking ligand expression of PD‐1, PD‐L1.^3^, ^7^, ^8^ Therefore, over half of ALM patients exclude from the benefits of BRAF‐, c‐Kit‐ and PD‐1‐targeted therapy. Thus, new molecular signatures of acral melanomas are needed to explore for more effective target therapy among ALM patients in future.

Recently, OPN3 (Opsin 3 or encephalopsin), which is the superfamily of heptahelical G protein‐coupled receptors and serves a variety of nonvisual functions,^9^, ^10^, ^11^ has been of interest in human epidermal melanocytes. Previous studies demonstrated that human OPN3 was widely expressed in various types of tissues such as skin, lung, brain, liver and testes, also known as panopsin.^9^, ^12^, ^13^ Recently we and others have found that OPN3 is highly expressed compared with other opsins (including OPN1SW, OPN2, OPN4, OPN5) in human epidermal melanocytes.^14^, ^15^, ^16^, ^17^ These studies demonstrated that OPN3 mediates light‐independent functions such as melanogenesis (pigmentation) and apoptosis in human epidermal melanocytes.^15^, ^16^

In tumors, recently reports showed that OPN3 gene was upregulated in pulmonary carcinoid tumors that developed postsurgical metastasis,^18^ and OPN3 promoted the epithelial‐mesenchymal transition and metastasis in lung adenocarcinoma.^19^ In addition, OPN3 depletion induced the 5‐fluorouracil resistance in hepatocellular carcinoma cells by activating the antiapoptotic pathway.^20^ However, its expression and role in cutaneous melanoma remain uncharacterized.

On the basis of these promising findings, along the line of the ALM initiation and development from benign pigmented macule to malignant primary melanoma to lymph node metastasis, we performed the expression of OPN3 in junctional melanocytic nevi (JMN) and ALMs on the foot, and inguinal lymph node metastasis (ILNMs) from foot ALMs, as well as its association with clinicopathological features and prognosis of ALMs.

## 2 METHODS

### 2.1 Sample selection and data collection

All the subjects of JMN (*n* = 12), ALMs (n = 39) and ILNMs (n = 23) were collected at Affiliated Hospital of Guizhou Medical University and Affiliated Cancer Hospital of Guizhou Medical University from January 2014 to December 2019. Immunohistochemistry (IHC)‐ and hematoxylin‐eosin‐stained sections were reviewed by an experienced pathologist, and cases fulfilling criteria for the appropriate diagnoses (JMN, ALM, and ILNM) were selected for study. In addition, five pairs of fresh tissues of ALMs on the foot and corresponding ILNMs from five patients were collected to verify the OPN3 expression by Western blot and quantitative real‐time PCR (qRT‐PCR). All five subjects provided written informed consent. All samples of JMN and ALM occurred on the foot, and ILNMs arose from the ALMs on the foot. The follow‐up period ended November 30, 2020. The study was approved by the Ethics Committees of the institution (Affiliated Hospital of Guizhou Medical University; Approval: #2019‐184) and was performed according to the Declaration of Helsinki.

### 2.2 IHC analyses of OPN3 expression

Section (4 μm) of formalin‐fixed, paraffin‐embedded tissues of JMN (*n* = 12), ALMs (*n* = 39), and ILNMs (*n* = 23) were dewaxed and rehydrated using standard methods.^21^ Antigen retrieval was performed in retrieval solution (ethylenediaminetetraacetic acid [EDTA], ZLI‐9069; ZSGB‐BIO) with a pH of 9.0 for 4 min using the pressure cooker antigen repairing method. The slides were treated with dual endogenous enzyme block 3% H_2_O_2_ (PV‐9000; ZSGB‐BIO, Beijing) to quench the endogenous peroxidase activity, and then a serum‐free blocking solution (ZLI‐9056; ZSGB‐BIO, Beijing) was used. Subsequently, the slides were incubated with anti‐OPN3 rabbit polyclonal antibody (MD4034‐100; Medical Discovery Leader (MDL), Beijing) at a dilution of 1:100 at 4°C overnight, followed by treatment with the 2‐step plus® Poly‐horseradish peroxidase (HRP) anti‐mouse/rabbit immunoglobulin G [IgG] Detection System (PV‐9000; ZSGB‐BIO, Beijing) according to the manufacturer's specifications. Color development and contrast were carried out with DAB Kit (ZLI‐9017; ZSGB‐BIO) and hematoxylin, respectively.

Two independent investigators scored all stained slides. The semiquantitative assessment was calculated using percentages of 3 + (strong), 2 + (moderate), 1 + (weak), and 0 (negative) staining of tumor cells for each case. The overall score was obtained by the percentage of positive malignant melanocytes (3 × x % + 2 × x % + 1 × x % = total score) to equal a range of 0–300.^22^

### 2.3 Multiple immunofluorescence staining

Thirty‐six representative specimens including all JMN (*n* = 12), part of ALMs (*n* = 12), and ILNMs (*n* = 12) among IHC‐positive staining tissues were sampled to perform the multi‐immunofluorescence assays. Formalin‐fixed paraffin‐embedded slices (4‐µm thickness) were deparaffinized and antigen unmasked. Then the specimens were blocked with 10% donkey serum at room temperature (RT) for 30 min and were incubated with first primary antibody of anti‐OPN3 (1:100; MD4034‐100; MDL) overnight at 4°C and secondary antibody marked with HRP incubate at RT for 50 min in the dark condition. Next, the sections were stained with cyanine 3 (CY3)‐tyramide signal amplification (TSA) solution (all from Siwega) at a 1:500 dilution for 10 min at RT in the dark. The slides were again immersed antigen retrieval buffer (EDTA) via microwave antigen repair method. Repeat the above steps, the slides were stained with the second primary antibody of anti‐Melan A (rabbit 1:1000; ab210546; Abcam), corresponding secondary antibody and CY5‐TSA (all from Siwega), respectively. DAPI (C0060; Solarbio) incubation at a 1:1000 dilution at RT for 5 min was used for nuclei staining.

### 2.4 qRT‐PCR assay

qRT‐PCR was performed as described previously.^15^ Total RNA was isolated from tumor tissues by TRIzol reagent (15596026; Invitrogen). FastKing‐RT SuperMix kit (KR118; TIANGEN) was used to synthesize first‐strand complementary DNA from 1.5 μg total RNA. qRT‐PCR analysis was carried out using an Eppendorf system (Realplex) with SYBR Green PreMix (FP209; TIANGEN) in the amplification reaction mixtures (25 μl). The primer sequences were: OPN3: 5′‐CAATCCAGTGATTTATGTCTTCATGATCAGAAAG‐3′ (forward); 5′‐GCATTTCACTTCCAGCTGCTGGTAGGT‐3′ (reverse); glyceraldehyde 3‐phosphate dehydrogenase: 5′‐GACATCCGCAAAGACCTG‐3′ (forward), 5′‐GGAAGGTGGACAGCGAG‐3′ (reverse). The reaction was taken by pre‐degeneration at 95°C for 10 min, 40 cycles at 95°C for 15 s, 60°C for 1 min. Relative messenger RNA (mRNA) level was calculated using the 2^−∆CT^ method.

### 2.5 Western blotting (WB)

Total protein extracts were obtained by tissue lysis in RIPA lysis buffer (R0010; Solarbio) containing 1 mM phenylmethylsulfonyl fluoride (R0010; Solarbio). For WB, 40 μg protein was separated by sodium dodecyl sulfate‐polyacrylamide gel electrophoresis and then transferred onto polyvinylidene difluoride (PVDF) membranes (Immobilon‐P; Millipore). Membranes were blocked with 5% nonfat milk for 2 h at room temperature and then soaked in primary antibodies overnight at 4°C. The OPN3 (1:500; MD4034‐100; MDL) and β‐Tubulin (1:1000; T2003; Affinity Biosciences) were used as primary antibodies. After washing four times with Tris‐buffered saline Tween washing buffer (TBST buffer), the blots were incubated with HRP‐conjugated anti‐mouse (1:1000; MD912524; MDL) or anti‐rabbit IgG (1:2000, BS912565; MDL) for 2 h at room temperature. After the PVDF membrane was washed three times with TBST, expression of the above proteins was detected using ECL WB detection reagent (7sea Biotechnology). The expression levels were measured with FastStone Image Viewer 5.5 software.

### 2.6 Statistical analyses

All data were entered into the GraphPad Prism (version 8.0) and IBM SPSS Statistics version 26 software program for statistical analysis. Continuous variables were summarized as means with *SD* or median with interquartile range (IQR) when distribution was skewed. Categorical data were described as counts and percentages. Data normality and equality of variances were analyzed using Shapiro–Wilk and Bartlett's tests, respectively. We used *t* tests and one‐way analysis of variance to compare means of two or more than two groups. Mann–Whitney (when comparing two groups) and the Kruskal–Wallis (more than two groups) tests were used to compare the nonparametric distributions. Survival analyses were made between patients with high and low expression of OPN3 via the Kaplan–Meier method.^23^ Multivariate Cox regression models were used to estimate adjusted hazard ratios (HRs) and 95% confidence intervals (CIs) for outcomes. A two‐tailed *p* < .05 was considered statistically significant.

## 3 RESULTS

### 3.1 Clinicopathologic data

Clinicopathologic characteristics of 62 cancer samples are highlighted in Table 1. The mean age at diagnosis among all 55 patients was 61.6 years, and 27 were male and 28 were female. Of the seven patients offered two samples including ALM and corresponding ILNM tissues. While the mean age of JMN cases was 43.7 years, with a ratio of male to female of 1:1. All patients were Chinese, and only accepted surgical treatment. Four of 62 samples (6.5%) harbored the *BRAF* ^*V600E*^ mutation, two of which were from the same patient. No mutations in *BRAF* ^*V600E*^ were detected in 12 JMN samples. The most common cell morphology observed in cancer groups was epithelioid (54.8%), followed by spindle cells (27.4%) (Figure S1 and S1).

**Table 1:** Patient clinicopathological characteristics of ALMs

Variable	*n* (%)
Total patients	55
Total samples	62
Sex	
Male	27 (49.1)
Female	28 (50.9)
Age (years, mean ± *SD*)	61.6 ± 12.4
<65	27 (49.1)
≥65	28 (50.9)
Tumor site	
Sole	30 (48.4)
Nail bed	9 (14.5)
Inguinal lymph node metastasis	23 (37.1)
Breslow depth (mm)	
0.01–1.00	8 (20.5)
1.01–2.00	3 (7.7)
2.01–4.00	11 (28.2)
>4.00	16 (41.0)
Unknown	1 (2.6)
Clark level	
I	0 (0)
II	4 (10.3)
III	7 (17.9)
IV	11 (28.2)
V	15 (38.5)
Unknown	2 (5.1)
Ulceration	
Present	32 (82.0)
Absent	6 (15.4)
Unknown	1 (2.6)
Morphologic features	
Epithelioid cells	34 (54.8)
Spindled cells	17 (27.4)
Pleomorphic cells	7 (11.4)
Plasmacytoid cells	3 (4.8)
Rhabdoid cells	1 (1.6)
*BRAF* ^*V600E*^ mutation	
Yes	4 (6.5)
No	58 (93.5)
Months survived after diagnosis	
Dead	
<12 months	17 (30.9)
≥12 months, ≤48 months	7 (12.7)
Alive	18 (32.7)
Unknown	13 (23.6)

*Note: BRAF* ^*V600E*^ status was previously assessed by direct sequencing (Sanger) in all cases.

Abbreviation: ALM, acral lentiginous melanoma.

### 3.2 Increasing expression of OPN3 from benign JMN to ALMs

**Figure 1:**
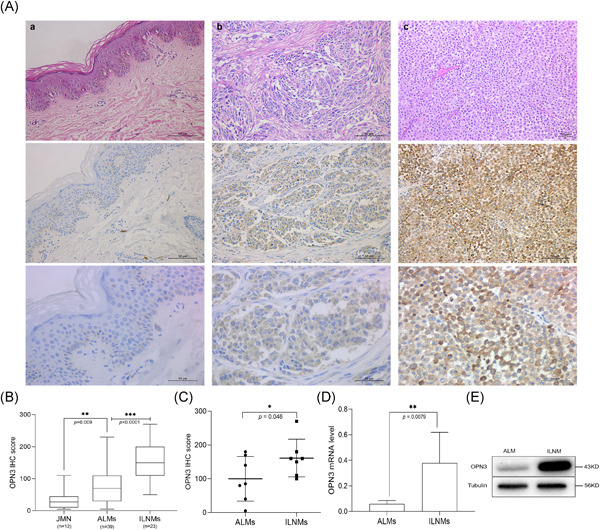
A. Immunohistochemistry findings of OPN3 of representative cases from JMN (A) to ALM (B) to ILNM (C) (HE, ×20 magnification; IHC, ×20, ×40 magnification). (B) Box‐and‐whiskers plot shows that the OPN3 immunohistochemistry score differs significantly between JMN and acral ALMs. Significance between the two groups was determined by the Mann–Whitney *U* test. The black lines inside the boxes are the median values for each group. The vertical size of the boxes is the interquartile range. The vertical “whiskers” represent the values range. **p < .01, ***p < .001. (C) The OPN3 score of immunohistochemical staining in seven paired samples of ALMs and ILNMs. *p < .05. (D) Expression levels of OPN3 in primary ALMs tissues (*n* = 5) and paired ILNMs tissues (*n* = 5) detected by qRT‐PCR (D) and WB (representative case, E). OPN3 mRNA expression in ILNMs tissues was significantly higher than primary ALMs tissues. Data represent mean ± *SD* of OPN3 levels normalized to that of GAPDH. OPN3 protein level was analyzed by WB with anti‐OPN3 antibody and Tubulin. **p < .01. ALM, acral lentiginous melanoma; GAPDH, glyceraldehyde 3‐phosphate dehydrogenase; HE, hematoxylin and eosin stain; ILNM, inguinal lymph node metastasis; JMN, junctional melanocytic nevi; mRNA, messenger RNA; WB, Western blotting

**Figure 2:**
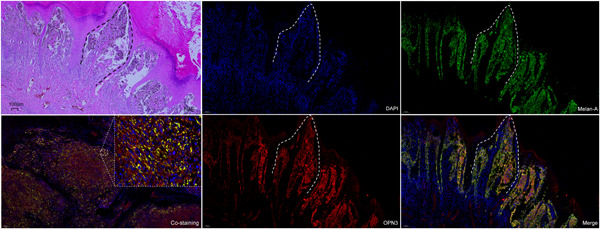
Immunofluorescence analysis of ALMs costained for Melan‐A (green) and OPN3 (red) proteins. Selected costaining image on the bottom left of panel displaying OPN3 expression mainly in cancer cells (orange) at low magnification and high‐magnification. The rest of panels showing OPN3 positive expression in tumor nests (dotted line) from HE to multi‐immunofluorescence. Scale bars indicate 100 µm. ALM, acral lentiginous melanoma; HE, hematoxylin and eosin stain

**Figure 3:**
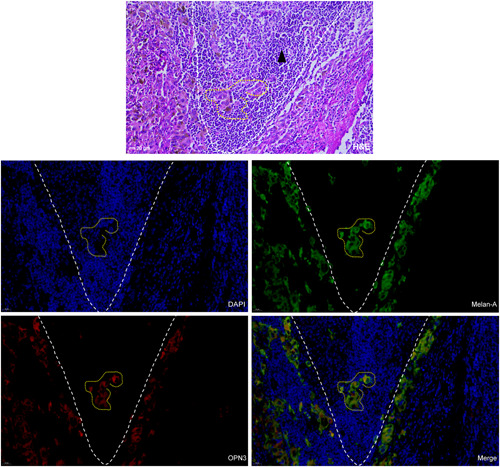
HE staining and multi‐immunofluorescence analysis of representative case from ILNM. The cancer cells (yellow dotted line) were positive by costaining of Melan‐A (green) and OPN3 (red) instead of lymphocytes (black triangle). Scale bars indicate 20 µm. HE, hematoxylin and eosin stain; ILNM, inguinal lymph node metastasis

OPN3 immunohistochemical staining revealed that six cases of JMN (6/12, 50%）, 8 cases of ALMs (8/39, 20.5%) and 0 case of ILNMs were negative or weak staining with an overall score of ≤20. The median OPN3 staining scores differed significantly among JMN (Median [IQR]) (27.50 [10.00, 45.00]), ALMs (70.00 [30.00, 102.50]) and ILNMs (150.00 [110.00, 200.00]) (*p* < .001) (Figure 1A,B). The median OPN3 score was the highest in ILNMs, while a lowest in JMN groups. Also, the seven paired samples from the same patient showed increase OPN3 expression along progression from primary ALMs to ILNMs (*p* = .046) (Figure 1C). To further identify the status of OPN3 in ALMs, we detected OPN3 mRNA and protein level in five pairs of fresh ALMs and ILNMs tissues using qRT‐PCR and WB assays. As shown in Figure 1D, OPN3 of ILNMs had a higher expression as compared to primary ALMs. Then, to assess whether OPN3 expression occurs in tumor cells, we determined that OPN3 was predominantly expressed in cancer cells by costaining of Melan‐A (Figure 2A) instead of stromal cells and lymphocytes (Figure 3).

### 3.3 Association between OPN3 and histopathologic variables of ALMs

OPN3 scores were analyzed against clinicopathological variables, and the presence of ulceration, increased Breslow thickness and greater Clark level were significantly correlated with OPN3 (Table 2). According to the Breslow depth divided into ≤2.0 mm, 2.01–4.0 mm, and >4.0 mm groups, the OPN3 scores were compared (*p* = .042). A statistically significant different was noted while comparing the score among ≤3 versus ≥4 Clark level (*p* = .012). We also estimated the associations OPN3 expression between lymphovascular invasion, perineural invasion, and tumor infiltrative lymphocytes in ALMs. High OPN3 score was associated with presence of perineural invasion, increased tumor infiltrative lymphocytes (*p* = .0272 and *p* = .0144, respectively). While there was not statistical significance in the presence and absence of lymphovascular invasion (*p* = .2364). In addition, no difference was found in other histopathologic features including age, gender, and *BRAF* ^*V600E*^ mutation. The mean OPN3 score was the highest in cases with predominant pleomorphic cells while spindle cells group was the lowest, however, there was no statistical significance among different cell types in cancers (*p* = .461) (Table 2).

**Table 2:** Comparison of OPN3 scores with clinicopathological variables in ALMs

Variable	Mean or median OPN3 staining score	
	Primary ALMs group	ILNMs group
Sex (mean ± *SEM*)		
Male	84.00 ± 14.77	154.10 ± 20.77
Female	74.21 ± 15.20	150.00 ± 15.72
	*p* value **=** .647	*p* value **=** .875
Age (years) (mean ± *SEM*)		
<65	89.74 ± 12.45	154.60 ± 17.99
≥65	69.25 ± 16.68	147.80 ± 17.06
	*p* value = .335	*p* value = .797
Tumor site (mean ± *SEM*)		
Sole	81.33 ± 12.85	N/A
Nail bed	72.22 ± 16.03	N/A
	*p* value = .719	N/A
Breslow depth (mm) (mean ± *SEM*)		
≤2.00	48.64 ± 11.00	N/A
2.01–4.00	61.82 ± 16.67	N/A
>4.00	107.20 ± 18.96	N/A
	*p* value = .042*	N/A
Clark level (median [IQR])		
I–III	30 [10.00, 70.00]	N/A
IV–V	80 [40.00, 147.50]	N/A
	*p* value = .012*	N/A
Ulceration (median [IQR])		
Present	80 [32.5, 230.0]	N/A
Absent	30 [10.0‐60.0]	N/A
	*p* value = .025*	N/A
Lymphovascular invasion (mean ± *SEM*)		
Present	92.78 ± 16.99	N/A
Absent	67.62 ± 12.76	N/A
	*p* value = .2364	
Perineural invasion (mean ± *SEM*)		
Present	111.50 ± 21.80	N/A
Absent	63.08 ± 10.25	N/A
	*p* value = .0272*	
Tumor infiltrative lymphocytes (mean ± *SEM*)		
Inactivity	58.26 ± 11.09	N/A
Activity	109.4 ± 17.78	N/A
	*p* value = .0144*	
Morphologic features (including primary ALMs and ILNMs) (mean ± *SEM*)		
Epithelioid cells	96.76 ± 10.94	
Spindled cells	102.10 ± 19.50	
Pleomorphic cells	134.30 ± 34.01	
	*p* value = .461	
*BRAF* ^*V600E*^ mutation (mean ± *SEM*)		
Yes	116.7 ± 52.39	
No	107.30 ± 9.42	
	*p* value = .828	
Months survived after diagnosis (mean ± *SEM*)		
Dead		
<12 months	145.60 ± 19.69	
≥12 months, ≤48 months	104.30 ± 14.62	
	*p* value = .216	
Alive	73.89 ± 14.10	
Dead	133.5 ± 14.92	
	*p* value = .007**	

Abbreviations: ALM, acral lentiginous melanoma; ILNM, inguinal lymph node metastasis; IQR, quartiles; N/A, not applicable; *SEM*, standard error of mean.

* *p* < .05

** *p* < .01.

### 3.4 Survival analysis

**Figure 4:**
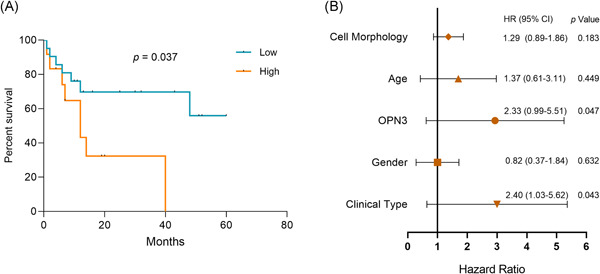
(A) Overall survival curve of ALMs patients with different staining score of OPN3 (<100 vs. ≥100) using the Kaplan–Meier method, and multivariate Cox regression models used to estimate adjusted hazard ratios (HRs) and 95% confidence intervals (CIs) for survival outcomes (B). Clinical type: primary ALMs and metastatic ALMs (ILNMs). Cell morphology: epithelioid cells, spindled cells, pleomorphic cells, plasmacytoid cells and rhabdoid cells. ALM, acral lentiginous melanoma; ILNM, inguinal lymph node metastasis

There was significant difference in average OPN3 scores between alive group and dead group among ALMs patients (*p* = .007). OPN3 expression did not differ significantly between patients who did not survived 12 months (*n* = 17) after diagnosis and patients who were alive 12 months or larger (*n* = 7) (*p* = .525). Furthermore, we conducted the prognostic analysis between OPN3 expression and survival status of the patients by log‐rank tests. The Kaplan–Meier survival analysis showed that a notable difference (*p* = .037) of patient overall survival was found between ≥100 and <100 immunohistochemical score of OPN3 groups (Figure 4A). Next, HRs and 95% CIs by Cox regression analysis showed that increased OPN3 expression (HR = 2.33 [95% CI: 0.99–5.51], *p* = .047) and presence of ILNM (HR = 2.40 [95% CI: 1.03–5.62], *p* = .043) were associated with worse overall survival in ALMs (Figure 4B). Given the incomplete follow‐up data was limited in our study, we further explored publicly available gene expression dataset (GSE98394)^24^ of primary cutaneous melanoma (*n* = 51), and survival analyses were made between patients with high and low expression of OPN3 via the Kaplan–Meier method.^23^ In the external dataset of cutaneous melanoma (GSE98394), we observed a significant difference in the level of OPN3 gene expression that affected patient overall survival between the upper 50% group versus other 50% group (*p* = .014) (Figure S2), which suggested that the upregulation of the OPN3 expression is associated with poor disease outcome of cutaneous melanoma.

## 4 DISCUSSION

Although its incidence is low, ALM accounts for approximately 50% of all melanomas in Asian populations.^6^, ^25^, ^26^ The prognosis of ALM at the advanced stages continues to remain dismal as compared to other types of cutaneous melanoma because of biologically aggressive even in their early T1 stage and delayed diagnosis.^4^, ^27^ In addition, the positive benefits of current first‐line treatments are limited on the ALM patients, both typical radiochemotherapy and molecularly targeted therapy.^3^, ^6^, ^7^, ^8^ Therefore, significant efforts have been made to explore other molecular subtypes that might impact patient care, to greatly improve the clinical outcomes of melanoma patients.^28^, ^29^ Interestingly, Ozdeslika et al recently identified a light‐independent function for OPN3 in the regulation of the melanogenic pathway in human epidermal melanocytes, by controlling the activity of the main pigmentation receptor, melanocortin 1 receptor (MC1R).^16^ Our study found that the downregulation of OPN3 induces apoptosis of human epidermal melanocytes by a calcium‐dependent G protein‐coupled signaling and mitochondrial pathway.^15^ Thus, not only OPN3 is expressed and affects apoptosis in human epidermal melanocytes, but also OPN3 is linked to the metastasis and drug resistance in some types of lung tumors and liver cancer, respectively.^15^, ^16^, ^17^, ^18^, ^19^, ^20^ For these reasons, we sought to investigate the clinicopathologic data and OPN3 expression patterns for ALMs to determine any associations with prognosis.

We observed a trend toward higher OPN3 immunohistochemical staining scores from begin JMN to malignant ALMs to ILNMs, which first indicated to us that OPN3 is a significant association with progression and metastasis of ALMs. The mRNA level of OPN3 expression between five paired ALMs and ILNMs fresh tissues has also proved it. Consequently, our results are consistent with those found in OPN3 expression of lung cancers,^18^, ^19^ and indicate that OPN3 might be a key molecule regulating metastasis of ALMs. Although the role of OPN3 in the pathogenesis of ALMs remain largely unclear, previous our and other studies found that OPN3 is involved in melanocyte apoptosis pathway,^15^ and interact with MC1R,^16^ which are the important pathway or key molecule in melanoma initiation and development.^30^ We also observed that the upregulation of OPN3 expression promoted the invasion of MV3 melanoma cells in vitro by transwell invasion assay (unpublished data). These studies indicated that aggressive biological behavior of ALM is related to high expression of OPN3. Moreover, significant difference was seen in OPN3 scores among ulceration, Breslow depth and Clark levels. Previous studies have demonstrated that age, ulceration, and increased tumor thickness are poor prognostic factors for ALM.^4^, ^31^ Hence, our findings suggested that OPN3 might be a novel prognostic indicator for ALMs.

Furthermore, we demonstrated that patients with high OPN3 protein expression level (above the 100 score) notably showed a poor prognosis in this study by survival analyses of the Kaplan–Meier and Cox regression models. And we offered additional evidence that the upregulation of the OPN3 gene expression in cutaneous melanoma is also associated with a poor disease outcome base on the gene expression dataset of GSE98394.^24^ These results were similar to the previous report of OPN3 expression correlated with overall survival in lung adenocarcinoma patients.^19^ Of note, OPN3 may serve as the target for therapy of melanoma in future. Therefore, it is important that future studies completely elucidate the molecular basis of association between OPN3 and melanoma.

## 5 CONCLUSIONS

We characterized the features of OPN3 expression among JNMs and ALMs as well as the association with clinicopathological factors and prognosis. In addition, we provided an important clue for OPN3 as a potential indicator for assessment of ALM prognosis. A larger study of OPN3 in ALMs may be greater powered to identify an association with ALM prognosis.

## CONFLICT OF INTERESTS

The authors declare that there are no conflict of interest.

## AUTHOR CONTRIBUTIONS

Study design: Hongguang Lu, Wen Zeng, Wei Zhang. Data collection: Jianglong Feng, Wen Zeng, Wei Zhang, Xiaoyan He. Data analyses: Wei Zhang, Wen Zeng, Jianglong Feng, Xiaoyan He, Hongguang Lu. Results interpretations: All authors. Manuscript writing: Wei Zhang, Hongguang Lu. Manuscript proofing: Hongguang Lu.

## Supporting information

Supplementary information.

## Data Availability

The data used to support the findings of this study are available from the corresponding author upon request.
