# Supplementary material for: Expression of OPN3 in acral lentiginous melanoma and its associated with clinicohistopathologic features and prognosis
Source: Immun Inflamm Dis. 2021 May 6;9(3):840–50. doi: 10.1002/iid3.438 (PMC8342238; doi:10.1002/iid3.438)
Supplement: Supplementary file 1 — Supplementary information. [file IID3-9-840-s001.docx]

**Figure S1**. Different cancer cell morphologic features (A-E) via H&E staining in ALMs ( H&E, ×40 magnification).

**Figure S2.** Overall survival (OS) curve of cutaneous melanoma patients based on the GSE98394 dataset with different expression level of OPN3 (Upper 50% *VS.* Other 50%; Upper 30% *VS.* Other 70%). e.g. Upper 50% (green): the cases with ranked top 50% higher expression level of the OPN3 gene; Other 50% (red): the cases with ranked bottom 50% lower expression level of OPN3 gene.
